# Supplementary material for: The causal relationship between the human gut microbiota and pyogenic arthritis: a Mendelian randomization study
Source: Front Cell Infect Microbiol. 2024 Nov 26;14:1452480. doi: 10.3389/fcimb.2024.1452480 (PMC11629706; doi:10.3389/fcimb.2024.1452480)
Supplement: Supplementary file 1 [file DataSheet1.zip › mendelian test/ebi-a-GCST90027523.csv_leave_one_out.pdf]

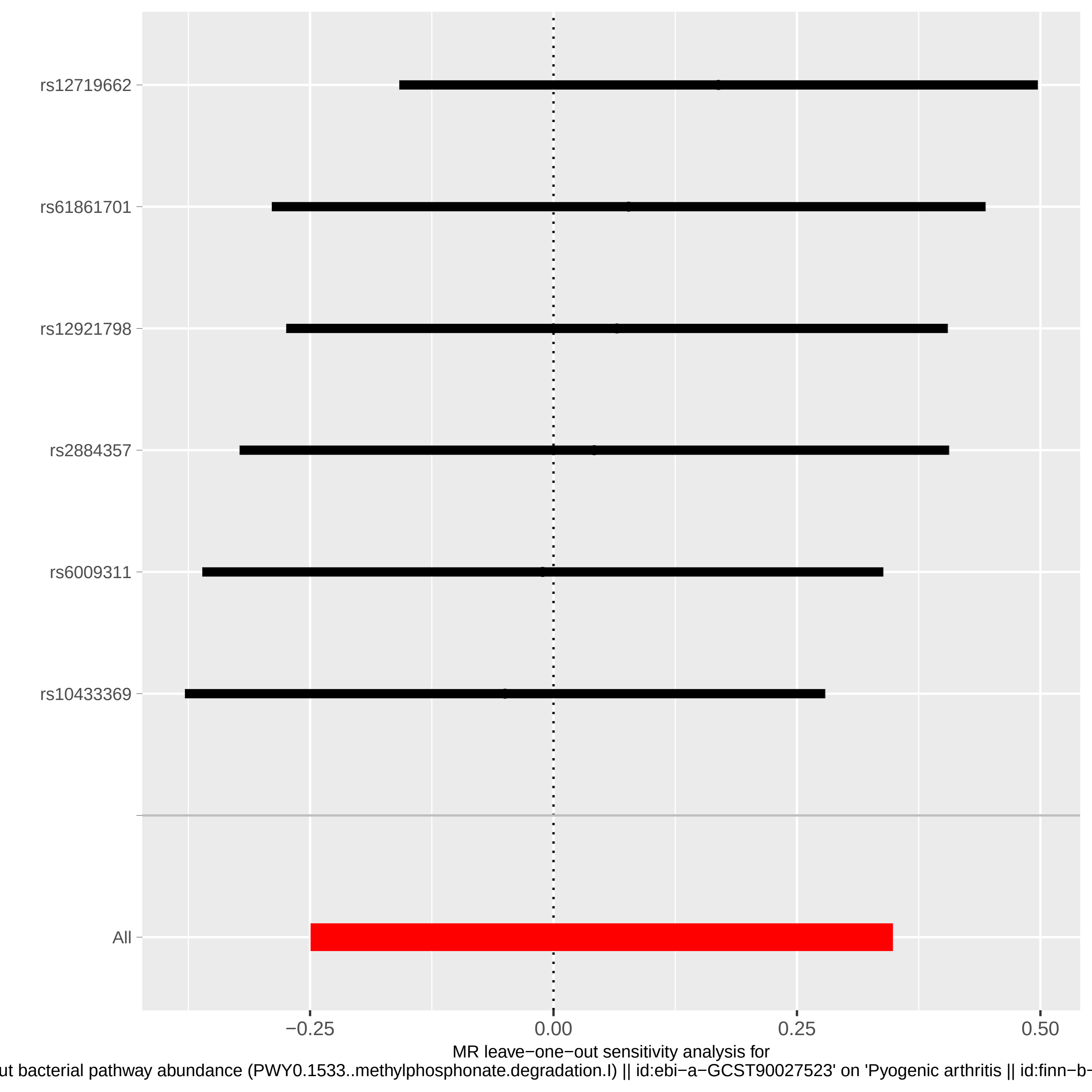
rs12719662
rs61861701
rs12921798
rs2884357
rs6009311
rs10433369
All
−0.25
0.00
0.25
0.50
MR leave−one−out sensitivity analysis for
ut bacterial pathway abundance (PWY0.1533..methylphosphonate.degradation.I) || id:ebi−a−GCST90027523' on 'Pyogenic arthritis || id:finn−b−
